# Clinical Evaluation of Ripasudil for Corneal Edema: A Large-Scale Retrospective Cohort Study

**DOI:** 10.3390/jcm14155572

**Published:** 2025-08-07

**Authors:** Nir Erdinest, Michael Tabi, Nadav Shemesh, Jamel Corredores, Claudia Yahalom, Yossi Eshel, Benjamin Stern, David Smadja, Zvi Gur, Itay Lavy

**Affiliations:** 1 Department of Ophthalmology, Hadassah University Medical Center, Faculty of Medicine, The Hebrew University of Jerusalem, Jerusalem 91904, Israel; nadavshemesh91@gmail.com (N.S.); jamelcdieb@gmail.com (J.C.); cyahalom@gmail.com (C.Y.); yosilach1@gmail.com (Y.E.); bystern@gmail.com (B.S.); smadj.david@gmail.com (D.S.); zvigur90@gmail.com (Z.G.); itaylavy@gmail.com (I.L.); 2 Faculty of Medicine, The Hebrew University of Jerusalem, Jerusalem 91904, Israel; michaeltabi92@gmail.com

**Keywords:** Ripasudil, corneal edema, Rho-associated protein kinase inhibitor, endothelial dysfunction, topical therapy

## Abstract

**Objectives:** This study evaluated the therapeutic potential of topical Ripasudil hydrochloride hydrate in managing various forms of corneal edema. **Methods:** This retrospective study included 96 patients of 72.20 ± 10.52 years, with 53 females (55.2%) who were treated with Ripasudil for corneal edema, with a mean treatment duration of 5.2 ± 2.3 months, divided into four groups: post-cataract surgery (n = 32), Fuchs endothelial corneal dystrophy (FECD; n = 29), post-Descemet membrane endothelial keratoplasty (DMEK; n = 25), and post-penetrating keratoplasty (PKP; n = 10). All patients were treated with Ripasudil, typically administered three times daily in the first week and twice daily in the following months. Clinical efficacy outcomes were assessed using changes in best-corrected visual acuity (BCVA), central corneal thickness (CCT), and endothelial cell count (ECC) with specular microscopy, anterior segment optical coherence tomography (OCT), and slit-lamp examination, while intraocular pressure (IOP) was measured using the iCare tonometer. **Results:** Ripasudil treatment led to a reduction in CCT and improvement in visual acuity across most groups, with minimal changes in ECC. CCT decreased by 30.44 μm (*p* < 0.001), 25.56 μm (*p* < 0.001), 8.41 μm (*p* = 0.05), and 6.80 μm (*p* > 0.1); visual acuity improved by 0.27 (*p* = 0.001), 0.18 (*p* = 0.02), 0.17 (*p* = 0.025), and 0.07 logMAR units (*p* > 0.1); and ECC changed by +7.0 (*p* > 0.1), 15.4 (*p* > 0.1), −7.6 (*p* > 0.1), and 2.3 cells/mm^2^ (*p* > 0.1) in the post-cataract surgery, FECD, post-DMEK, and post-PKP groups, respectively. **Conclusions:** No adverse events or progression of edema were recorded during the follow-up period. These findings support the role of Ripasudil as a non-invasive pharmacological approach to managing corneal edema and delaying or possibly avoiding surgical interventions, such as corneal transplantation, in selected cases.

## 1. Introduction

The accumulation of fluid in the corneal stroma [1,2] is a defining feature of corneal edema, leading to significant visual compromise due to reduced corneal transparency. It originates primarily from pathological states, such as Fuchs endothelial corneal dystrophy (FECD) [1,2,3], post-procedural complications, and traumatic ocular injury. This pathological condition exhibits heterogeneous severity, compromising visual function to variable degrees. The corneal endothelium assumes a fundamental role in preserving stromal dehydration and maintaining optical transparency [1,4].

Endothelial cell count (ECC), expressed as the number of endothelial cells per mm^2^, serves as a vital indicator of posterior corneal integrity [1,4]. In individuals aged 20–39, ECC typically averages around 3000 cells/mm^2^ and declines steadily at a rate of 0.3%–0.6% annually. By the age of 60–79, average values decrease to approximately 2600 cells/mm^2^, with counts below 1000–1200 cells/mm^2^ associated with increased risk of corneal decompensation [5,6]. A reduction in ECC is often accompanied by an increase in central corneal thickness (CCT), due to impaired endothelial function and fluid imbalance [5,7]. CCT refers to the distance between the anterior corneal epithelium and the posterior endothelial surface at the corneal apex. It does not represent the total corneal thickness, which gradually increases toward the periphery. In healthy adults, the average CCT is approximately 550 μm, with peripheral values up to 23% greater due to natural corneal geometry [7]. Interindividual variation has been reported in the range of 520–570 μm [7].

Although corneal transplantation remains the primary treatment approach for advanced corneal edema, alternative strategies such as therapeutic contact lenses [8], pharmacological agents [1,9,10], and minimally invasive procedures like collagen cross-linking are being adopted with growing frequency [11,12]. These evolving modalities focus on maintaining corneal deturgescence, with treatments like topical hypertonic saline often employed, despite mixed evidence regarding their efficacy. Advances in surgical technique and postoperative care have reduced the incidence of corneal edema following cataract surgery, a cornerstone of ophthalmic practice [13,14,15]. Nevertheless, complications can still arise, including mechanical trauma, inflammation, chemical injuries, and worsening of underlying conditions [13,14,15]. The variable incidence of postoperative corneal edema underscores the importance of tailored patient assessment and management [14,16,17,18]. Ripasudil, a Rho-associated protein kinase (ROCK) inhibitor approved in Japan for the treatment of glaucoma and ocular hypertension, enhances aqueous outflow via the trabecular meshwork and has shown additional potential in supporting corneal endothelial function [19,20]. Since this pharmacological approach focuses on reducing intraocular pressure (IOP), its mechanism has prompted growing interest in its potential use for corneal edema, where it may support endothelial function and promote cytoskeletal remodeling to facilitate fluid resolution [21,22].

Alternative ROCK inhibitors such as Netarsudil, which was approved in the US and Europe, and Fasudil, which was approved in Japan, offer distinct therapeutic alternatives, each possessing unique characteristics and regulatory approvals across various geographical areas [21,22]. Current clinical investigations continue to examine the therapeutic potential of novel ROCK inhibitors for treating multiple ocular disorders. While Ripasudil shows promise due to its pharmacological mechanism of action, comprehensive clinical evidence confirming its efficacy across various forms of corneal edemas in real-world settings is still limited [19,20]. Standard dosing typically requires administration three times daily in the first week and twice daily in the following weeks and may lead to adverse effects such as blepharitis.

This study aims to assess the efficacy of Ripasudil in the management of diverse corneal edema etiologies through a retrospective analysis conducted at a single tertiary center. Given that endothelial keratoplasty remains the current standard of care, the study further explores the urgent need for effective non-surgical treatment alternatives for corneal edema.

## 2. Materials and Methods

### 2.1. Ethical Considerations

Institutional Review Board (IRB) approval was obtained from Hadassah Medical Center (0418-22HMO, December 2022), and all procedures adhered strictly to the approved protocol. This study was conducted in accordance with the Declaration of Helsinki and relevant Clinical Practice Guidelines. Informed consent was obtained from all participants prior to their enrollment.

### 2.2. Data Collection and Sources

Clinical data were systematically retrieved from the “Mahar” electronic medical records (EMR) system at Hadassah Medical Center. Patient identification was conducted through targeted queries supported by the institution’s Business Intelligence (BI) unit. Complementary imaging data were collected from the Spectralis Optical Coherence Tomography (OCT) system and the Picture Archiving and Communication System (PACS), both of which form essential components of the ophthalmic imaging infrastructure at Hadassah. Only patients with complete imaging datasets, including CCT and ECC measurements, were included in the study cohort to ensure data integrity and consistency.

### 2.3. Study Population

This retrospective study analyzed 96 patients who received Ripasudil treatment for corneal edema. The overall study population had a mean age of 72.20 ± 10.52 years, with 53 females (55.2%) and 43 males (44.8%). The distribution of affected eyes included 49 right eyes (51.0%) and 47 left eyes (49.0%). Patients were excluded if they had incomplete datasets, active ocular infections, or were using other corneal edema treatments (e.g., hypertonic saline). The mean duration of corneal edema prior to treatment was 1.69 ± 0.54 months for the post-cataract surgery group, 3.38 ± 3.30 months for the FECD group, 0.44 ± 0.51 months for the post-Descemet membrane endothelial keratoplasty (DMEK) group, and 7.7 ± 2.54 months for the post-penetrating keratoplasty (PKP) group.

### 2.4. Study Variables

The study incorporated both pre-treatment and post-treatment evaluations. Baseline assessments included measurements of best-corrected visual acuity (BCVA), CCT, and ECC. Information was gathered regarding the overall treatment duration, measured in months, and the frequency of DMEK procedures performed after initiating therapy.

### 2.5. Diagnostic Categories and Treatment Indications

Patients were categorized into four distinct diagnostic groups based on the underlying etiology of corneal edema and the clinical indication for Ripasudil treatment. These groups included patients with corneal edema following cataract surgery, patients diagnosed with FECD, patients presenting with corneal edema following DMEK, and patients with corneal edema following PKP. This distribution provides a comprehensive representation of the primary clinical scenarios where Ripasudil therapy is considered for corneal edema management, encompassing both acute post-surgical conditions and chronic endothelial pathologies.

### 2.6. Treatment Protocol

The therapeutic protocol consisted of topical instillation of Ripasudil hydrochloride hydrate (Glanatec^®^, 0.4% ophthalmic solution; Kowa Co., Ltd., Nagoya, Japan). The eye drops were administered three times each day during the initial week, followed by twice-daily applications in subsequent weeks. Adjustments in frequency were implemented as necessary based on clinical severity and in accordance with the manufacturer’s recommendations, with a mean treatment duration of 5.2 ± 2.3 months.

### 2.7. Assessment Parameters

Treatment efficacy evaluation was based on clinical assessments conducted both prior to and following administration of Ripasudil. Baseline measurements included BCVA, recorded using the logMAR scale, CCT measured with the Pentacam HR (Oculus, Wetzlar, Germany), and ECC assessed using specular microscopy with the Konan NonCon-ROBO instrument (Konan Medical USA, Inc., Irvine, CA, USA). Slit-lamp evaluations were conducted with the Haag-Streit BX 900 Photo-Slit Lamp system (Haag-Streit Diagnostics, a division of Haag-Streit AG, Koeniz, Switzerland), and anterior segment imaging was performed using spectral-domain optical coherence tomography with the Optovue RTVue-100 device (Visionix USA, formerly Optovue Inc., Fremont, CA, USA). Intraocular pressure (IOP) measurements were obtained using the iCare ic100 handheld tonometer (Icare Finland Oy, a subsidiary of Revenio Group, Vantaa, Finland).

Adverse event monitoring was conducted throughout the study period to ensure patient safety. Regular slit-lamp examinations were performed to assess for complications such as blepharitis, conjunctival hyperemia, or ocular surface discomfort. Patients were instructed to report any adverse symptoms, which were documented during follow-up visits. IOP was measured using the iCare ic100 handheld tonometer to monitor for potential IOP-related adverse events. All adverse events were recorded in the electronic medical records system and reviewed by the clinical team. No serious adverse events were reported during the study period, with mild ocular surface discomfort noted in a small subset of patients.

Follow-up evaluations post-treatment replicated the baseline tests and included documentation of treatment duration in months. Slit-lamp examinations and anterior segment OCT imaging were repeated and compared with pre-treatment findings.

### 2.8. Statistical Analysis

Statistical analyses were performed using SPSS software, version 25.0 (IBM Corp., Armonk, NY, USA). Patients with incomplete datasets were excluded to ensure data integrity, resulting in the exclusion of three patients due to missing imaging data or loss to follow-up. Missing data for primary outcomes (CCT, BCVA, ECC) were minimal (<5%), and no imputation was performed. The exclusions were attributed to absent ECC data, with 2 cases lacking the examination entirely and 1 case due to failure to obtain ECC readings from the specular microscopy device. All analyses were conducted on a per-protocol basis, with complete datasets available for 96 patients across all subgroups. Given the repeated measures design, a paired *t*-test was employed to compare pre- and post-treatment values across diagnostic groups, while an independent two-tailed t-test was used to evaluate differences in BCVA, CCT, and ECC outcomes. Normality was assessed using the Shapiro–Wilk test, and homogeneity of variances was evaluated using Levene’s test. Non-parametric alternatives were used when assumptions were violated. One-way ANOVA was used for between-group comparisons of CCT and ECC when normality assumptions were met, and the Kruskal–Wallis test was used for BCVA due to non-normality in the PKP group, with post hoc pairwise comparisons adjusted using Bonferroni correction. The Bonferroni correction was applied to adjust for multiple comparisons, including gender-based subgroup comparisons and between-group analyses across the four diagnostic groups and three primary outcomes (CCT, BCVA, ECC), setting the corrected significance threshold. In small subgroups (e.g., PKP, n = 10), Shapiro–Wilk tests were supplemented with visual inspections using box plots to confirm data distribution, as shown in Supplementary Figure S1. Multivariable linear regression models were used to assess treatment effects on CCT, BCVA, and ECC, adjusting for age, gender, and duration of corneal edema prior to treatment, implemented using SPSS. All analyses adhered to variance assumptions, and statistical significance was defined at *p* < 0.05. Ninety-five percent confidence intervals (95% CI) were calculated for all primary outcomes using standard error–based methods. Sample size determination followed a continuous outcome model with two independent groups, targeting a 95% CI and a statistical power of 80%. Based on these parameters, the estimated minimum sample size required to detect a meaningful difference was post-cataract surgery patients, who formed the largest cohort, followed by FECD, post-DMEK, and PKP. The limited sample size within the PKP group may have restricted statistical power.

Statistical analysis was conducted by gender, with female participants comprising 53 individuals and male participants comprising 43 individuals across the four diagnostic groups. The primary outcomes examined included CCT measured in micrometers, BCVA measured in logMAR units, and ECC measured in cells per square millimeter, with all measurements assessed as changes from baseline to post-treatment values. For each outcome measure within each diagnostic group, mean changes were calculated by subtracting pre-treatment values from post-treatment values, accompanied by standard deviation calculations performed separately for male and female patient cohorts. Statistical significance was evaluated through independent two-sample t-tests comparing mean changes between genders within each diagnostic group. All *p*-values were interpreted in light of the Bonferroni-adjusted thresholds where applicable. *p*-value for BCVA in the PKP group was calculated using the non-parametric Wilcoxon rank-sum tests due to non-normal distribution.

## 3. Results

### 3.1. Demographics and Group Distribution

A total of 99 patients were initially identified, and 3 were excluded due to missing imaging data or loss to follow-up. The final analysis included 96 patients with complete datasets.

The post-cataract surgery, FECD, post-DMEK, and PKP groups consisted of 32, 29, 25, and 10 patients, respectively. The mean ages were 70.5 ± 10.93, 74.24 ± 8.97, 72.36 ± 9.62, and 64.7 ± 4.24 years, respectively (58–97, 57–89, 56–94, and 59–70 years). The gender distribution included 20 females (62.5%) and 12 males (37.5%), 19 females (65.5%) and 10 males (34.5%), 9 females (36.0%) and 16 males (64.0%), and 5 females (50.0%) and 5 males (50.0%) for the post-cataract surgery, FECD, post-DMEK, and PKP groups, respectively.

The patients were distributed across four diagnostic groups: edema post-cataract surgery, FECD, corneal edema post-DMEK, and corneal edema after PKP (Table 1).

In addition, full descriptive statistics, including means, standard deviations, medians, ranges, and 95% confidence intervals for all outcomes at baseline and follow-up across subgroups, are presented in Supplementary Table S2.

### 3.2. Ripasudil Treatment Duration

The duration of Ripasudil treatment varied across groups. Patients with edema post-cataract surgery received treatment for 5.03 ± 2.66 months. Those with FECD were treated for 4.97 ± 1.95 months. For corneal edema post-DMEK, the treatment durations were 5.48 ± 2.02 months, while patients with corneal edema after PKP underwent treatment for 5.60 ± 2.91 months.

### 3.3. Clinical Outcomes by Group

#### 3.3.1. Post-Cataract Surgery Group

The post-cataract surgery group demonstrated the most significant therapeutic response to Ripasudil treatment. CCT showed a substantial reduction from 599.91 ± 23.10 μm to 569.47 ± 20.64 μm, with a mean decrease of 30.44 μm (95% CI: −41.61 to −19.27, *p* < 0.001). Visual acuity improved significantly from 0.55 ± 0.45 logMAR to 0.28 ± 0.36 logMAR, with a mean improvement of 0.27 logMAR units (95% CI: −0.48 to −0.06, *p* = 0.001). ECC remained stable, showing a minimal non-significant decrease from 963.16 ± 183.53 to 970.19 ± 187.88 cells/mm^2^ (95% CI: −87.66 to 101.72, *p* > 0.1).

Multivariable regression analyses, adjusting for age, gender, and edema duration, confirmed these findings, with significant reductions in CCT (β = −28.12 µm, *p* < 0.001) and BCVA (β = −0.24 logMAR, *p* = 0.002) and non-significant ECC changes (β = 6.8 cells/mm^2^, *p* > 0.1) (Supplementary Table S3).

#### 3.3.2. Fuchs Endothelial Corneal Dystrophy Group

Patients with FECD exhibited significant corneal thickness reduction, with CCT decreasing from 638.59 ± 24.03 μm to 613.03 ± 19.54 μm, with a mean reduction of 25.56 μm (95% CI: −37.34 to −13.78, *p* < 0.001). Visual acuity improved, decreasing from 0.60 ± 0.54 logMAR to 0.42 ± 0.49 logMAR, with a mean improvement of 0.18 logMAR units (95% CI: −0.46 to 0.10, *p* = 0.02). ECC demonstrated minimal change from 737.61 ± 169.66 to 722.24 ± 175.40 cells/mm^2^, with a decrease of 15.4 cells/mm^2^ (95% CI: −121.27 to 91.03, *p* > 0.1).

#### 3.3.3. Corneal Edema Post-DMEK Group

The post-DMEK group showed improvements in corneal parameters. CCT decreased from 630.57 ± 26.47 μm to 622.16 ± 21.57 μm, with a mean reduction of 8.41 μm (95% CI: −15.15 to −0.85, *p* = 0.05, not significant after Bonferroni correction). Visual acuity demonstrated improvement, changing from 0.53 ± 0.48 logMAR to 0.36 ± 0.40 logMAR, with a mean improvement of 0.17 logMAR units (95% CI: −0.35 to −0.01, *p* = 0.025, not significant after Bonferroni correction). ECC remained essentially unchanged from 849.44 ± 169.41 to 841.88 ± 170.36 cells/mm^2^, with a decrease of 7.6 cells/mm^2^ (95% CI: −77.42 to 61.50, *p* > 0.1).

#### 3.3.4. Corneal Edema After PKP Group

The PKP group, despite having the highest baseline CCT values, showed minimal response to treatment. CCT decreased marginally from 665.90 ± 45.37 μm to 659.10 ± 53.03 μm, with a mean reduction of 6.80 μm (95% CI: −38.88 to 25.08, *p* > 0.1). Visual acuity showed minimal improvement from 0.66 ± 0.68 logMAR to 0.59 ± 0.71 logMAR, with a mean improvement of 0.07 logMAR units (95% CI: −0.59 to 0.45, *p* > 0.1). ECC remained stable at 1113.00 ± 232.48 versus 1110.70 ± 346.86 cells/mm^2^, with a decrease of 2.3 cells/mm^2^ (95% CI: −217.77 to 212.57, *p* > 0.1).

**Figure 1:**
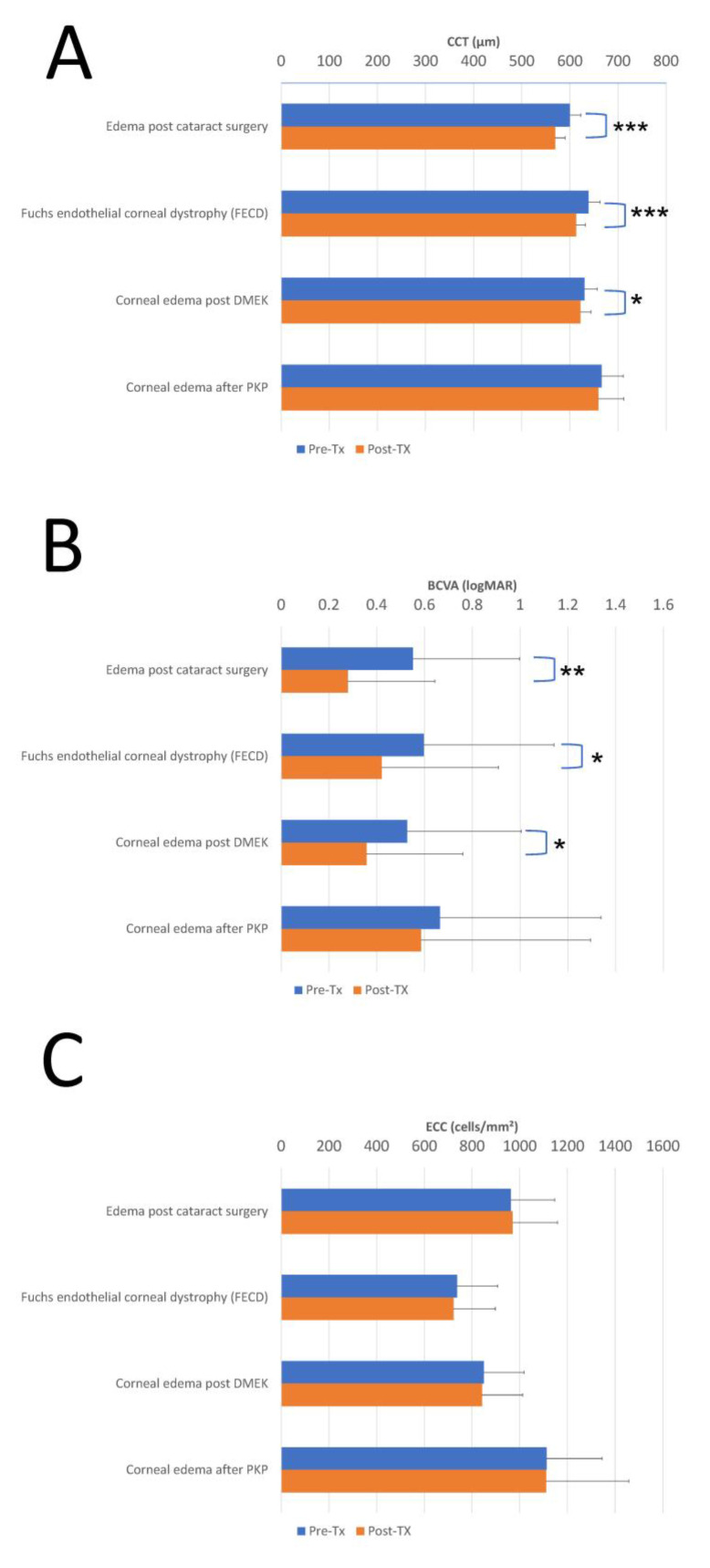
Parameter changes pre- and post-treatment in four corneal edema subgroups. Bar graphs illustrate the mean ± standard deviation values of CCT (μm), BCVA (logMAR), and ECC (cells/mm^2^) in pre- and post-treatment conditions. The x-axes represent absolute measured values for each parameter, and error bars represent standard deviation. (**A**) Central corneal thickness (CCT) demonstrates changes in CCT, defined as the distance between the anterior epithelial surface and the posterior endothelial surface. The reduction in CCT following treatment represents therapeutic resolution of corneal edema across all groups. (**B**) Best-corrected visual acuity presents visual acuity outcomes expressed in logarithm of the minimum angle of resolution (logMAR) units. (**C**) Endothelial cell count demonstrates changes in corneal endothelial cell density, measured as cells per square millimeter. CCT: central corneal thickness, BCVA: best-corrected visual acuity, ECC: endothelial cell count, FECD: Fuchs endothelial corneal dystrophy, DMEK: Descemet membrane endothelial keratoplasty, PKP: penetrating keratoplasty. Asterisks adjacent to the post-treatment bars indicate the level of statistical significance. * represents a statistically significant difference with *p*  <  0.05, ** indicate *p*  <  0.01, and *** indicate *p*  <  0.001.

These changes across all four diagnostic subgroups are visually summarized in Figure 1.

#### 3.3.5. Between-Group Comparisons

One-way ANOVA revealed significant differences in CCT reduction across groups (*p* < 0.001), with post hoc Bonferroni-corrected tests (*p* < 0.0083) showing greater reductions in the post-cataract surgery (30.44 µm, *p* < 0.001) and FECD groups (25.56 µm, *p* < 0.001), compared to the post-PKP group. The Kruskal–Wallis test for BCVA showed significant differences (*p* = 0.003), with post hoc tests indicating greater improvement in the post-cataract surgery group (0.27 logMAR, *p* = 0.001), compared to the post-PKP group. No significant differences were observed for ECC (ANOVA *p* > 0.1) (Supplementary Table S3).

#### 3.3.6. Responder Analyses

Responder analyses were conducted to assess clinically meaningful outcomes, defining responders as patients achieving ≥20 µm reduction in CCT or ≥0.1 logMAR improvement in BCVA. In the post-cataract surgery group, 71.9% (23/32) and 68.8% (22/32) achieved these thresholds for CCT and BCVA, respectively. In the FECD group, 65.5% (19/29) and 58.6% (17/29) met these thresholds. Lower rates were observed in the post-DMEK (36.0%, 9/25 for CCT; 32.0%, 8/25 for BCVA) and post-PKP groups (20.0%, 2/10 for both) (Supplementary Table S3).

#### 3.3.7. Sensitivity Analyses

Sensitivity analyses were performed by excluding patients with extreme values (BCVA > 2 logMAR or CCT > 700 µm) to assess result robustness. These analyses confirmed consistent findings, with a CCT reduction of 29.12 µm (*p* < 0.001) and BCVA improvement of 0.25 logMAR (*p* = 0.001) in the post-cataract surgery group and similar stability in the FECD (CCT 24.10 µm, *p* < 0.001; BCVA 0.16 logMAR, *p* = 0.02), post-DMEK, and post-PKP groups (Supplementary Table S3).

### 3.4. IOP Differences Among Groups

IOP measurements before and after treatment with ROCK inhibitors were compared across four clinical subgroups. In the group with corneal edema following cataract surgery, mean IOP decreased from 13.59 ± 2.87 mmHg to 13.28 ± 2.53 mmHg (95% CI: −1.34 to 0.72, *p* = 0.28). Among patients with FECD, IOP changed from 12.62 ± 2.66 mmHg to 12.21 ± 2.04 mmHg (95% CI: −1.42 to 0.60, *p* = 0.33). In the DMEK group, the IOP was 12.8 ± 1.89 mmHg before treatment and 12.6 ± 1.83 mmHg after treatment (95% CI: −0.98 to 0.58, *p* = 0.61). In eyes with corneal edema after PKP, IOP decreased from 12.2 ± 2.35 mmHg to 11.9 ± 1.45 mmHg (95% CI: −1.98 to 1.38, *p* = 0.42).

### 3.5. Gender-Stratified Analysis of Ripasudil Effectiveness

To investigate potential gender differences in Ripasudil’s effectiveness, a stratified analysis was conducted for the primary outcomes (CCT, BCVA, ECC) across the four diagnostic groups. In the post-cataract surgery group (n = 32; 12 females, 20 males), females exhibited a mean CCT reduction of 30.58 ± 22.67 µm, compared to 30.35 ± 24.02 µm in males (*p* = 0.927). BCVA improved by 0.29 ± 0.45 logMAR in females and 0.25 ± 0.44 logMAR in males (*p* = 0.672), while ECC changes were minimal (5.58 ± 39.25 cells/mm^2^ in females vs. 8.75 ± 47.55 cells/mm^2^ in males, *p* = 0.824). In the FECD group (n = 29; 19 females, 10 males), males showed a slightly greater CCT reduction (27.30 ± 22.93 µm vs. 24.79 ± 29.92 µm, *p* = 0.742), but BCVA and ECC changes were comparable between genders (*p* = 0.978 and *p* = 0.615, respectively). For the post-DMEK group (n = 25; 9 females, 16 males), no notable gender differences were observed, with CCT reductions of 7.33 ± 22.97 µm in females and 9.06 ± 29.83 µm in males (*p* = 0.774), BCVA improvements of 0.18 ± 0.39 logMAR in females and 0.14 ± 0.49 logMAR in males (*p* = 0.615), and ECC changes of −6.22 ± 60.59 cells/mm^2^ in females vs. −8.94 ± 73.44 cells/mm^2^ in males (*p* = 0.896). In the post-PKP group (n = 10; 5 females, 5 males), gender differences were minimal, with CCT changes of −8.60 ± 34.06 µm in females vs. −5.00 ± 65.00 µm in males (*p* = 0.741), BCVA changes of −0.08 ± 0.19 logMAR in females vs. −0.06 ± 0.83 logMAR in males (*p* = 0.879, Wilcoxon rank-sum test), and ECC changes of 2.60 ± 76.16 cells/mm^2^ in females vs. 2.00 ± 280.79 cells/mm^2^ in males (*p* = 0.985). All *p*-values exceeded the Bonferroni-corrected threshold (*p* < 0.00417), indicating no significant gender-specific differences in Ripasudil’s effectiveness. Detailed results are presented in Supplementary Table S1.

## 4. Discussion

This study demonstrates the variable therapeutic efficacy of Ripasudil across different causes of corneal edema, with greatest therapeutic gains seen in patients with post-cataract surgery corneal edema. The differential response patterns observed across diagnostic groups provide important insights into the potential mechanisms of action and optimal clinical applications of this ROCK inhibitor. As mentioned, the post-cataract group showed the most significant response, suggesting greater efficacy in inflammation-related edema. The degree of reduction in corneal thickness and enhancement in visual acuity represents clinically meaningful changes that would be readily apparent to both patients and clinicians. Similarly, the FECD group demonstrated significant corneal thickness reduction along with significant visual acuity improvement, highlighting the therapeutic potential of Ripasudil in reducing corneal edema in patients with endothelial dysfunction and providing meaningful visual benefits despite the chronic nature of the underlying pathology. Multivariable regression analyses further support Ripasudil’s efficacy, with significant CCT and BCVA improvements in the post-cataract surgery (β = −28.12 µm, *p* < 0.001; β = −0.24 logMAR, *p* = 0.002) and FECD groups (β = −23.45 µm, *p* < 0.001; β = −0.16 logMAR, *p* = 0.018) after adjusting for age, gender, and edema duration, reinforcing the robustness of these findings (Supplementary Table S3).

The reductions in CCT and improvements in BCVA observed in this study have notable clinical significance. These values exceed commonly accepted thresholds for clinical significance, typically defined as a ≥20–30 μm reduction in CCT or ≥0.1 logMAR gain in BCVA. In the post-cataract surgery group, a mean CCT reduction of 30.44 μm (*p* < 0.001) likely contributes to improved corneal transparency and patient comfort, reducing symptoms such as glare and blurred vision. Similarly, the 0.27 logMAR improvement in BCVA (*p* = 0.001) corresponds to a gain of approximately 2–3 lines on a Snellen chart. In the FECD group, a 25.56 μm reduction in CCT and 0.18 logMAR improvement in BCVA suggest that Ripasudil can provide functional benefits, despite the chronic nature of endothelial dysfunction. Responder analyses further confirm clinical significance, with 71.9% (23/32) and 68.8% (22/32) of post-cataract surgery patients and 65.5% (19/29) and 58.6% (17/29) of FECD patients achieving ≥20 µm CCT reduction and ≥0.1 logMAR BCVA improvement, respectively, potentially delaying surgical interventions (Supplementary Table S3). These improvements may delay the need for surgical interventions, such as corneal transplantation, in selected patients, enhancing quality of life and reducing treatment burden. However, interpretation of these results requires careful consideration of potential confounding factors and sources of bias that may influence the observed therapeutic response across diagnostic groups. In the post-cataract surgery group, the natural resolution of postoperative inflammation, dry eye, and edema may have contributed to the observed improvements in CCT and BCVA, potentially confounding the therapeutic effect of Ripasudil, since cataract surgery-induced corneal edema often demonstrates spontaneous improvement over weeks to months as inflammatory mediators subside. Similarly, in the post-DMEK group, the healing process following endothelial keratoplasty likely played a role in corneal deturgescence, making it challenging to isolate Ripasudil’s specific contribution given the close temporal proximity between the procedure and treatment initiation. Additional confounding factors include baseline differences in endothelial function, variations in treatment adherence, and differences in the duration of corneal edema prior to treatment. Although no multivariable adjustment was performed, the influence of these confounding variables was considered in the interpretation of outcomes. The retrospective design introduces selection bias, particularly due to the lack of a control group, and the smaller sample size in the PKP group may limit statistical power and generalizability. These factors underscore the need for cautious interpretation of the results and highlight the importance of prospective, controlled studies to confirm Ripasudil’s efficacy.

Posterior stromal ripples have emerged as a clinically relevant biomarker for predicting visual recovery and graft stability following DMEK [23,24]. Posterior stromal ripple undulations in the posterior stroma seen in imaging are thought to reflect structural stress at the graft–host interface, potentially impairing optical quality and visual outcomes [23,25]. Recent studies have found that PSRs correlate with delayed visual recovery and a higher risk of graft detachment and re-bubbling [23,24,26]. Ventura et al. showed that patients with preoperative posterior stromal ripples had significantly slower visual improvement and worse final acuity [23]. Similarly, Lohmann et al. and Coco et al. identified posterior stromal ripples as risk indicators for detachment and poor graft adherence [23,25]. Structural analyses by Kilian et al. further supported this, linking ripples with topographic instability at the posterior surface [26]. While some authors such as Parekh et al. and Levis et al. emphasized that not all ripples impair vision, some being transient or benign, they still advocate for better classification systems based on posterior stromal ripples severity and persistence [24,27].

From a mechanistic perspective, the preservation of ECC across all treatment groups is encouraging and indicates that Ripasudil does not exert deleterious effects on corneal endothelial cells, which reinforces the drug’s safety profile while suggesting that its primary mechanism of action likely involves enhancement of existing endothelial pump function and cytoskeletal activity rather than cellular regeneration or proliferation. Sensitivity analyses excluding extreme values (BCVA > 2 logMAR or CCT > 700 µm) corroborate these findings, with consistent CCT reductions (29.12 µm, *p* < 0.001) and BCVA improvements (0.25 logMAR, *p* = 0.001) in the post-cataract surgery group, enhancing confidence in Ripasudil’s efficacy across diverse etiologies, though the small PKP group size limits statistical power (Supplementary Table S3). However, it is important to note that while ECC stability may imply preserved endothelial function, it should not be interpreted as evidence of cellular proliferation. The regenerative implications should therefore be viewed cautiously, particularly in the absence of statistically significant ECC increases. The ROCK inhibition pathway may enhance endothelial barrier function and pump activity through effects on cytoskeletal dynamics and intercellular junction integrity, which would explain the observed reduction in corneal thickness without corresponding changes in cell count. From a clinical standpoint, the variable response across diagnostic groups indicates that Ripasudil may be most beneficial as an adjunctive therapy in acute or subacute corneal edema conditions, notably those with an inflammatory component. The limited response in the PKP group, though possibly impacted by the small sample size, may also reflect the chronic nature of post-keratoplasty edema and the presence of more severe endothelial dysfunction.

Therapeutics for corneal edema have advanced over time, incorporating therapeutic contact lenses [8], with pharmacological modalities gaining increasing prominence alongside surgical interventions such as corneal transplantation. The evolving interest in ROCK inhibitors such as Ripasudil for the management of corneal edema is grounded in the historical limitations of conventional topical therapies. As reviewed by several studies, medical treatments for corneal edema, including hypertonic saline, corticosteroids, and bandage contact lenses, have traditionally offered only modest and often transient symptomatic relief, primarily targeting epithelial hydration rather than addressing the underlying endothelial dysfunction [1,10]. The limited efficacy of these approaches, particularly in cases of chronic or endothelial-derived edema, underscores the unmet therapeutic need that has prompted investigation into agents capable of modulating cellular function at a deeper level. ROCK inhibitors, by enhancing endothelial barrier integrity and pump activity, represent a paradigm shift in this regard, offering a mechanism-based intervention aligned with the core pathophysiology of stromal swelling and endothelial compromise. Topical hypertonic saline has traditionally been used to manage corneal edema, although its effectiveness remains weakly supported by empirical evidence. Its mechanism relies on increasing tear film osmolality to facilitate corneal dehydration [1,10]. Several factors, including endothelial integrity and IOP regulation, contribute to sustaining optimal corneal hydration homeostasis. Disruption of these mechanisms can lead to the onset of corneal edema [9].

Cataract extraction is a fundamental ophthalmologic intervention that significantly improves quality of life for millions worldwide [13]. Recent advances in operative techniques and postoperative management have improved visual outcomes and corneal integrity [14,15]. The prevalence of corneal edema or decompensation after cataract surgery ranges from 0.2% to 2.4% [14]. Corneal edema subsequent to intracapsular cataract extraction with anterior chamber or iris-fixated intraocular lens insertion occurs at significantly higher rates compared to intracapsular cataract extraction (ICCE) without intraocular lens (IOL) placement. Post-cataract surgery corneal edema develops from four primary causes [14,15]. First, mechanical trauma during the procedure is a predominant factor, often attributable to ultrasonic energy or instrument contact, underscoring the intrinsic procedural risks [14,15]. Second, inflammation or infection-related complications may play a role, often triggered by retained nuclear material or inadequately treated infections, requiring immediate therapeutic intervention [14,16,17,18]. Third, chemical injuries may result from intraoperative substances, emphasizing the importance of meticulous surgical preparation. Finally, pre-existing pathologies such as FECD may potentiate postoperative edema, necessitating comprehensive preoperative assessment for optimal patient management [16,17,18].

Ripasudil is a selective Rho-associated protein kinase inhibitor developed by Kowa Company and approved in Japan for glaucoma and ocular hypertension. Its mechanism involves enhancement of aqueous humor efflux through the trabecular meshwork, receiving regulatory approval for use in treatment-resistant cases [19,20]. The primary therapeutic goal in glaucoma and ocular hypertension management is IOP normalization [19,20]. Ripasudil has recently demonstrated potential as a therapeutic option for ocular pathologies, including corneal edema, by modulating cellular contractility and enhancing endothelial cell functionality to support resolution of edema. This activity is mediated through inhibition of the Rho/ROCK signaling pathway, which regulates actomyosin contraction, tight junction formation, and cytoskeletal organization in endothelial and trabecular meshwork cells. Ripasudil acts downstream of various G protein-coupled receptors, inhibiting the Rho/ROCK signaling cascade that mediates cytoskeletal tension, cell adhesion, and endothelial permeability [19,28,29]. By suppressing Rho kinase activity, Ripasudil reduces actin stress fiber formation and cellular stiffness, facilitating aqueous outflow and promoting a more permissive environment for endothelial repair [19,20]. However, clinical evidence validating its therapeutic effectiveness across diverse corneal edema manifestations, particularly in real-world clinical settings, remains limited [19,20]. The treatment protocol involves thrice-daily administration and may lead to adverse reactions, such as blepharitis [29].

Two additional ROCK inhibitors are commercially available: Netarsudil in the United States and Europe and Fasudil in Japan. Each agent is indicated for distinct therapeutic applications [21,22]. Netarsudil acts by selective inhibition of both ROCK1 and ROCK2 isoforms, enhancing trabecular meshwork drainage and reducing episcleral venous pressure. In preclinical studies, it has also shown neuroprotective effects on axons in rat models. Authorized in the United States in 2017 and Europe in 2019 for managing elevated IOP in primary open-angle glaucoma or ocular hypertension, Netarsudil is administered once daily [22,30,31]. The drug has demonstrated consistent IOP reduction throughout circadian cycles and maintained acceptable safety profiles across numerous clinical studies. Ripasudil and Netarsudil lack regulatory authorization in the United Kingdom, Canada, or Australia. Fasudil, marketed since 1995 in Japan, is primarily used for the treatment of cerebral vasospasm and has also been assessed for potential use in diabetic macular edema [22,29,30,31]. Other ROCK inhibitors, such as SNJ-1656 and Y-27632, are still undergoing clinical evaluation but have not yet been approved for commercial use [22,30,31]. Although Ripasudil, Netarsudil, and Fasudil share the same general mechanism as Rho kinase inhibitors, Ripasudil primarily enhances aqueous outflow through the trabecular meshwork, Netarsudil combines this effect with a reduction in episcleral venous pressure, and Fasudil exerts broader systemic vasodilatory actions, originally developed for cerebral vasospasm [32]. Importantly, differences in molecular selectivity, ocular penetration, and receptor affinity among these agents may explain their distinct clinical indications and dosing regimens [22,30,31].

The enhancement in BCVA observed in our study aligns with prior reports demonstrating notable visual gains in patients with FECD following Ripasudil therapy [20,33,34]. With regard to CCT, our results are consistent with those of a preceding case series in which Ripasudil was used for the treatment of segmental corneal edema, leading to partial or complete resolution of the edema in the majority of cases [35]. This receives further reinforcement from findings in a study that examined the incidence of persistent corneal edema post-cataract surgery, which emphasizes the therapeutic potential of Ripasudil for this indication [19,36]. Although the modest rise in ECC did not reach statistical significance, it still holds clinical significance. Prior research indicates that Ripasudil can upregulate gene and protein expressions involved in cell cycle regulation, cell–matrix adhesion, and cellular migration [19,36]. In addition, another study strongly advocated the use of ROCK inhibitors as adjunctive therapy in cataract surgery, particularly in more complex scenarios, such as cases involving FECD [19,36]. Lastly, in our cohort, the average duration of Ripasudil treatment was approximately 4.9 months, during which 29% of patients eventually required DMEK. This trend suggests that Ripasudil may contribute to postponing more invasive interventions like DMEK, a notion supported by earlier evidence in the literature.

A published case series described four instances of persistent corneal edema treated successfully with topical Ripasudil following anterior segment surgeries [35]. The underlying conditions included FECD, pseudophakic bullous keratopathy, and endothelial cell loss post-keratoplasty. Administered three times a day, Ripasudil induced both visual improvement and resolution of corneal edema, with no adverse effects reported [35]. These findings support Ripasudil’s potential as a safe and effective treatment option while also highlighting the need for further investigation into optimal dosing regimens and treatment durations [35].

An additional study explored Ripasudil’s regenerative effects on corneal endothelial cells in FECD patients, utilizing both ex vivo tissue and in vitro cell models. As mentioned, the results demonstrated that Ripasudil positively influences endothelial cell behavior, enhancing cell cycle activity, adhesion, and migration without altering the normal phenotype in either affected or unaffected endothelial cells [34]. Moreover, Ripasudil was found to augment the expression of proteins essential for maintaining endothelial pump function and barrier integrity. Nevertheless, these in vitro and ex vivo findings do not directly translate to clinical evidence of proliferation in vivo, and the observed benefits in our cohort are more plausibly attributed to functional enhancement of existing cells. Collectively, these findings strengthen the rationale for using ROCK inhibitors like Ripasudil as regenerative agents in the management of patients with FECD [34].

A clinical study evaluated the efficacy and safety of Descemet Stripping Only (DSO) combined with Ripasudil administration for managing FECD, including a total of 23 eyes [33]. All cases underwent DSO, followed by postoperative treatment with Ripasudil. Remarkably, corneal clearance was achieved in 22 out of 23 eyes within an average of 4.1 weeks, accompanied by significant improvement in BCVA. The study applied stringent inclusion criteria, selecting participants with a minimum superior ECC of 1000 cells/mm^2^ and central guttata as the primary cause of visual decline [33]. Safety monitoring was thorough, including adverse event reporting, regular blood pressure checks, and routine blood tests. No serious adverse effects were observed, with the most frequently reported side effects being mild ocular surface discomfort and gastrointestinal symptoms. In terms of visual outcomes, the study demonstrated a meaningful gain in uncorrected visual acuity, with mean logMAR improving from 0.43 preoperatively to 0.24 at nine months post-treatment [33].

Similarly, best spectacle-corrected BCVA showed a marked improvement, with the mean logMAR decreasing from 0.15 preoperatively to 0.002 at 12 months following surgery. ECC was assessed both before and after the procedure. Although a postoperative decline in superior ECC was noted, the comparison between the Ripasudil-treated group and the control group revealed no statistically significant difference [33].

Another study evaluated the effectiveness of Ripasudil in enhancing BCVA among patients with FECD [20]. A total of 30 eyes from 15 individuals were randomized to receive either Ripasudil treatment or serve as controls. The intervention group was administered 0.4% Ripasudil eye drops three times daily over an 18-month period [20]. Patients receiving Ripasudil experienced significant improvements in both BCVA and corneal edema [20]. Specular microscopy revealed a mean ECC of 727 ± 142 cells/mm^2^ in the treatment group. A variety of diagnostic modalities, including anterior segment OCT, were utilized to support the findings. The authors concluded that Ripasudil represents a promising therapeutic option for managing FECD [20].

In an additional case series, the combined use of Ripasudil therapy and femtosecond laser-assisted cataract surgery was examined in relation to corneal endothelial morphology [36]. In the first case, improvements were noted in endothelial cell shape and size in the operated eye, indicating the potential benefit of Ripasudil as an intraoperative adjunct. The second case demonstrated a reduction in corneal decompensation, confirmed through both topographic and confocal imaging. Collectively, these cases underscore Ripasudil’s role in enhancing endothelial cell morphology and mitigating edema. These findings further support the application of ROCK inhibitors in treating endothelial dysfunction and reinforce the importance of ongoing research in this area [36].

A recent study found that Ripasudil eye drops significantly reduced endothelial cell loss after cataract surgery, suggesting a protective effect on the corneal endothelium [37]. The findings indicated a lower degree of endothelial cell loss among patients treated with Ripasudil [19]. Additionally, no significant differences were observed between the groups with respect to age, sex, or preoperative corneal thickness [19]. These results suggest that Ripasudil may serve as a viable therapeutic approach for addressing low ECC post-cataract surgery, highlighting the necessity for further research to validate its efficacy [19].

This study found that Ripasudil did not promote an increase in ECC, implying that its therapeutic effect is more likely due to enhanced cellular function rather than stimulation of proliferation. These findings align with earlier studies that reported similar results [33,38,39]. Accordingly, the discussion of regenerative potential should remain tempered by the understanding that functional improvement can occur in the absence of increased cell density. The resolution of corneal edema may be explained by one of two mechanisms: either Ripasudil promotes cytoskeletal remodeling and cellular migration, leading to structural changes and the reopening of a previously non-functional endothelial defect once treatment is withdrawn, or it improves the performance of cells within an already established endothelial monolayer [33,38,39].

No statistically significant differences in IOP were observed between pre- and post-treatment in any of the groups. Despite Ripasudil’s known pharmacologic effect as an IOP-lowering agent, the absence of measurable change in this cohort suggests that the apparent IOP fluctuations during corneal edema and its resolution are more likely due to biomechanical alterations of the cornea rather than actual physiological changes [40]. During edema, increased corneal thickness and pliability reduce applanation resistance, leading to underestimation of true IOP values [41]. These findings support the hypothesis that tonometric variability, particularly under conditions of corneal edema, reflects measurement artifacts, rather than genuine changes in IOP.

Therefore, the observed IOP differences across groups should be interpreted with caution. For example, Neuburger et al. demonstrated that Goldmann tonometry systematically underestimates true intracameral pressure in edematous corneas, whereas rebound tonometers like iCare provide more accurate readings [41]. Similarly, studies of post-phacoemulsification corneal edema reported that Goldmann tonometry readings were several mmHg lower than rebound tonometry values [42,43,44]. As edema resolves and corneal structure normalizes, restoring thickness and rigidity, tonometric accuracy improves. Consequently, an apparent IOP rise following edema resolution often reflects restored measurement precision rather than a pathological increase in ocular pressure. Thus, variations in measured IOP may reflect biomechanical recovery rather than actual fluctuations. ROCK inhibition has also demonstrated vascular benefits in diabetic macular edema (DME), including reduction in central foveal thickness independent of IOP effects, likely through mechanisms such as stabilization of endothelial tight junctions and VEGF suppression [45].

The gender-stratified analysis revealed no statistically significant differences in Ripasudil’s effectiveness between male and female patients across the four diagnostic groups for CCT, BCVA, or ECC. This finding suggests that Ripasudil’s therapeutic benefits are consistent, regardless of gender, supporting its broad applicability in treating corneal edema. However, slight variations were observed, such as a marginally greater CCT reduction in females in the post-cataract surgery group and in males in the FECD group, though these differences were not statistically significant. The absence of gender-specific effects may be attributed to the similar baseline characteristics and disease mechanisms across genders, as well as the relatively small sample sizes, particularly in the PKP group (n = 5 per gender), which may limit statistical power. Future studies with larger cohorts are needed to confirm these findings and explore potential gender-related differences in treatment response, particularly in less common etiologies like post-PKP edema.

While this study offers valuable preliminary insights into the use of Ripasudil for the treatment of corneal edema, several limitations must be acknowledged. The relatively short follow-up period limits the ability to evaluate long-term safety and sustained therapeutic efficacy. In addition, the distribution of patients across the clinical subgroups was not balanced. This was particularly notable in the PKP subgroup, which included only a small number of patients, potentially reducing the statistical strength and generalizability of the findings; as such, the results should be interpreted as exploratory and hypothesis-generating. Moreover, the retrospective nature of the study, which addressed corneal edemas of various etiologies, did not allow for the inclusion of a formal control group. Although this design reflects real-world clinical practice, the addition of a control cohort would have enhanced the interpretability of the outcomes. Another limitation was the absence of a direct comparison with other pharmacological agents that have been shown to reduce corneal edema, such as Netarsudil or Fasudil. 

An additional consideration is the increased risk of type I error due to the multiple comparisons conducted across subgroups and clinical endpoints in this study. While Bonferroni correction was applied to account for multiplicity in between-group comparisons across diagnostic categories and primary outcomes (CCT, BCVA, ECC) and for gender-based subgroup comparisons, no formal correction was applied to the within-group pre- and post-treatment comparisons. This increases the likelihood of false-positive findings, particularly given the number of subgroup analyses and outcomes evaluated. Therefore, the observed statistically significant changes, especially those with marginal *p*-values or small effect sizes, should be interpreted with caution. As previously noted, the application of Bonferroni correction (*p* < 0.00417) for multiple comparisons across the four diagnostic groups and three primary outcomes (CCT, BCVA, ECC) ensures conservative interpretation, reducing type I error risk. Significant findings in the post-cataract surgery (CCT *p* < 0.001, BCVA *p* = 0.001) and FECD groups (CCT *p* < 0.001) persist, while non-significant results in the post-DMEK group (CCT *p* = 0.05, BCVA *p* = 0.025) may reflect limited statistical power (Supplementary Table S2). Global tests (ANOVA for CCT and ECC, Kruskal–Wallis for BCVA) confirm significant differences in treatment response across groups (CCT *p* < 0.001, BCVA *p* = 0.003), with post-cataract surgery and FECD groups showing superior outcomes compared to post-PKP, supporting targeted use of Ripasudil in acute and subacute edemas (Supplementary Table S3).

Given the promising results of this study, further research is warranted to address its limitations and confirm Ripasudil’s efficacy. Prospective, randomized controlled trials with larger sample sizes, extended follow-up periods, and comparator arms (e.g., Netarsudil or placebo) are recommended to validate these findings. Such studies should include balanced subgroup sizes, particularly for post-keratoplasty edema, and control for confounding factors such as natural recovery and baseline endothelial function. These efforts will help establish Ripasudil’s role as a non-invasive treatment for corneal edema and its potential to delay or avoid surgical interventions.

## 5. Conclusions

In synthesis, whereas endothelial keratoplasty remains the gold-standard therapeutic intervention, a critical need persists for alternative, non-invasive treatment modalities. This retrospective, observational study provides evidence supporting the potential use of Ripasudil in the management of corneal edema across various etiologies, with significant reductions in CCT and improvements in visual acuity observed in most subgroups. However, the study’s design introduces several limitations, including its observational nature, lack of a control group, unbalanced subgroup sizes, and relatively short follow-up period, which constrain the ability to draw definitive conclusions about Ripasudil’s efficacy. The documented improvements in pivotal ocular measurements correlate with existing scientific literature, accentuating Ripasudil’s therapeutic promise as an efficacious treatment modality. However, the absence of a significant change in ECC suggests that the benefits observed are more likely attributable to preserved or enhanced endothelial function rather than true regenerative activity. These findings highlight Ripasudil’s potential as an adjunctive therapy, particularly in post-cataract surgery and FECD-related edema, though further research is needed to confirm its long-term efficacy and optimal clinical applications. Specifically, future studies should employ extended follow-up periods to differentiate natural recovery from sustained Ripasudil effects. To enhance statistical power, larger sample sizes are needed, especially for the PKP group. Additionally, randomized controlled trials with appropriate controls would help mitigate confounding factors and confirm Ripasudil’s efficacy across corneal edema etiologies.

## Figures and Tables

**Table 1:** Demographic and clinical characteristics of patients treated with Ripasudil.

Characteristic	Value
**Patients (n)**	96
**Treated Eyes (n)**	R: 49, L: 47
**Age (years)**	72.2 ± 10.5 (range: 48–97)
**Gender**	Female: 53 (55.2%), Male: 43 (44.8%)
**Indication for Ripasudil (n, %)**	Post-cataract surgery: 32 (33.3%)
	FECD: 29 (30.2%)
	Post-DMEK edema: 25 (26.0%)
	Post-PKP edema: 10 (10.4%)

R: right eye; L: left eye; PKP: penetrating keratoplasty; DMEK: Descemet membrane endothelial keratoplasty; FECD: Fuchs endothelial corneal dystrophy.

## Data Availability

The datasets generated during and/or analyzed during the current study are available from the corresponding author upon reasonable request.

## Supplementary Materials

The following supporting information can be downloaded at: https://www.mdpi.com/article/10.3390/jcm14155572/s1. Table S1: Gender-Stratified Analysis of Ripasudil Effectiveness; Table S2: Comparison of Mean Values, 95% Confidence Intervals, and Ranges for IOP, CCT, Visual Acuity (logMAR), and Endothelial Cell Count Before and After Treatment Across Corneal Edema Etiologies; Table S3. Summary of Statistical Analyses for Ripasudil Treatment Effects Across Di-agnostic Groups; Figure S1: Visual assessments of data distribution in the PKP group (n = 10).
