# Supplementary material for: Clinical Evaluation of Ripasudil for Corneal Edema: A Large-Scale Retrospective Cohort Study
Source: J Clin Med. 2025 Aug 7;14(15):5572. doi: 10.3390/jcm14155572 (PMC12347265; doi:10.3390/jcm14155572)
Supplement: Supplementary file 1 [file jcm-14-05572-s001.zip › jcm-3750691-supplementary.pdf]

Supplementary Materials

The following supporting material can be accessed at: https://www.mdpi.com/article/doi/s1

Table S1. Gender-Stratified Analysis of Ripasudil Effectiveness

Diagnostic Group	Outcome	Gender	n	Mean Change (Post-Tx - Pre-Tx) ± SD	p-value
Post-Cataract Surgery	CCT (μm)	Female	12	-30.58 ± 22.67	0.927
		Male	20	-30.35 ± 24.02	
	BCVA (logMAR)	Female	12	-0.29 ± 0.45	0.672
		Male	20	-0.25 ± 0.44	
	ECC (cells/mm²)	Female	12	5.58 ± 39.25	0.824
		Male	20	8.75 ± 47.55	
Fuchs Endothelial Corneal Dystrophy (FECD)	CCT (μm)	Female	19	-24.79 ± 29.92	0.742
		Male	10	-27.30 ± 22.93	
	BCVA (logMAR)	Female	19	-0.17 ± 0.52	0.978
		Male	10	-0.17 ± 0.58	
	ECC (cells/mm²)	Female	19	-14.37 ± 36.12	0.615
		Male	10	-17.10 ± 49.58	
Post-DMEK	CCT (μm)	Female	9	-7.33 ± 22.97	0.774
		Male	16	-9.06 ± 29.83	
	BCVA (logMAR)	Female	9	-0.18 ± 0.39	0.615
		Male	16	-0.14 ± 0.49	
	ECC (cells/mm²)	Female	9	-6.22 ± 60.59	0.896
		Male	16	-8.94 ± 73.44	
Post-PKP	CCT (μm)	Female	5	-8.60 ± 34.06	0.741
		Male	5	-5.00 ± 65.00	
	BCVA (logMAR)	Female	5	-0.08 ± 0.19	0.879 *
		Male	5	-0.06 ± 0.83	
	ECC (cells/mm²)	Female	5	2.60 ± 76.16	0.985
		Male	5	2.00 ± 280.79	

CCT: Central Corneal Thickness, BCVA: Best-Corrected Visual Acuity, logMAR: Logarithm of the Minimum Angle of Resolution, ECC: Endothelial Cell Count, FECD: Fuchs Endothelial Corneal Dystrophy, DMEK: Descemet Membrane Endothelial Keratoplasty, PKP: Penetrating Keratoplasty, SD: Standard Deviation, Tx: Treatment.

Table S2. Comparison of Mean Values, 95% Confidence Intervals, and Ranges for IOP, CCT, Visual Acuity (logMAR), and Endothelial Cell Count Before and After Treatment Across Corneal Edema Etiologies

6	Phase	IOP Mean ± SD	IOP 95% CI	IOP Range	IOP Median	CCT Mean ± SD	CCT 95% CI	CCT Range	CCT Median	VA logMAR Mean ± SD	VA logMAR 95% CI	VA logMAR Range	VA logMAR Median	ECC Mean ± SD	ECC 95% CI	ECC Range	ECC Median
Edema post cataract surgery	Pre-Tx	1.69 ± 0.54	1.32 – 2.06	1.00 – 3.00	1.90	13.59 ± 2.87	11.60 – 15.58	10.00 – 21.00	14.86	599.91 ± 23.10	583.90 – 615.91	574.00 – 686.00	619.97	0.55 ± 0.45	0.24 – 0.86	0.08 – 2.48	1.04
	Post-Tx	963.16 ± 183.53	835.97 – 1090.34	554.00 – 1219.00	912.05	13.28 ± 2.53	11.53 – 15.03	10.00 – 19.00	14.09	569.47 ± 20.64	555.17 – 583.77	540.00 – 654.00	587.82	0.28 ± 0.36	0.03 – 0.53	0.00 – 2.00	0.76
Fuchs endothelial corneal dystrophy (FECD)	Pre-Tx	3.38 ± 3.30	1.09 – 5.67	1.00 – 12.00	5.46	12.62 ± 2.66	10.77 – 14.47	8.00 – 18.00	12.87	638.59 ± 24.03	621.93 – 655.24	607.00 – 730.00	658.53	0.60 ± 0.54	0.22 – 0.97	0.10 – 2.48	1.06
	Post-Tx	737.61 ± 169.66	620.04 – 855.18	396.00 – 1122.00	751.87	12.21 ± 2.04	10.79 – 13.62	9.00 – 18.00	13.07	613.03 ± 19.54	599.49 – 626.58	560.00 – 645.00	606.01	0.42 ± 0.49	0.08 – 0.76	0.00 – 2.00	0.81
Corneal edema post DMEK	Pre-Tx	0.44 ± 0.51	0.09 – 0.79	0.00 – 1.00	0.48	12.80 ± 1.89	11.49 – 14.11	10.00 – 16.00	12.93	630.57 ± 26.47	612.23 – 648.92	592.00 – 690.00	637.52	0.53 ± 0.48	0.20 – 0.86	0.10 – 2.50	1.04
	Post-Tx	849.44 ± 169.41	732.04 – 966.84	345.00 – 1085.00	759.81	12.60 ± 1.83	11.33 – 13.87	10.00 – 16.00	12.87	622.16 ± 21.57	607.21 – 637.11	569.00 – 669.00	620.05	0.36 ± 0.40	0.08 – 0.64	0.00 – 2.00	0.79
Corneal edema after PKP	Pre-Tx	7.70 ± 2.54	5.94 – 9.46	5.00 – 12.00	8.23	12.20 ± 2.35	10.57 – 13.83	8.00 – 16.00	12.07	665.90 ± 45.37	634.46 – 697.34	559.00 – 710.00	644.97	0.66 ± 0.68	0.20 – 1.13	0.10 – 2.52	1.10
	Post-Tx	1113.00 ± 232.48	951.90 – 1274.10	822.00 – 1490.00	1141.67	11.90 ± 1.45	10.90 – 12.90	9.00 – 14.00	11.63	659.10 ± 53.03	622.35 – 695.85	540.00 – 721.00	640.03	0.59 ± 0.71	0.09 – 1.08	0.05 – 2.50	1.05

IOP: Intraocular Pressure, CCT: Central Corneal Thickness, VA: Visual Acuity, logMAR: Logarithm of the Minimum Angle of Resolution, ECC: Endothelial Cell Count, DMEK: Descemet Membrane Endothelial Keratoplasty, PKP: Penetrating Keratoplasty, FECD: Fuchs Endothelial Corneal Dystrophy, Pre-Tx: Before Treatment, Post-Tx: After Treatment.

Table S3. Summary of Statistical Analyses for Ripasudil Treatment Effects Across Diagnostic Groups

Analysis	Diagnostic Group	Outcome	Result	p-value
Multivariable Regression	Post-Cataract Surgery	CCT (μm)	-29.85 (95% CI: 26.12–33.58)	<0.001
		BCVA (logMAR)	-0.26 (95% CI: 0.21–0.31)	0.001
		ECC (cells/mm²)	6.45 (95% CI: -12.34 to 25.24)	0.782
	FECD	CCT (μm)	-24.92 (95% CI: 20.45–29.39)	<0.001
		BCVA (logMAR)	-0.16 (95% CI: 0.11–0.21)	0.002
		ECC (cells/mm²)	-13.56 (95% CI: -35.12 to 8.00)	0.645
	Post-DMEK	CCT (μm)	-8.12 (95% CI: 4.56–11.68)	0.008
		BCVA (logMAR)	-0.15 (95% CI: 0.09–0.21)	0.012
		ECC (cells/mm²)	-7.12 (95% CI: -34.56 to 20.32)	0.891
	Post-PKP	CCT (μm)	-6.80 (95% CI: -21.45 to 7.85)	0.342
		BCVA (logMAR)	-0.07 (95% CI: -0.25 to 0.11)	0.465*
		ECC (cells/mm²)	2.30 (95% CI: -56.78 to 61.38)	0.972
Responder Analysis	Post-Cataract Surgery	BCVA (≥0.1 logMAR)	68.8% (22/32)	-
		CCT (≥20 μm)	71.9% (23/32)	-
	FECD	BCVA (≥0.1 logMAR)	58.6% (17/29)	-
		CCT (≥20 μm)	65.5% (19/29)	-
	Post-DMEK	BCVA (≥0.1 logMAR)	56.0% (14/25)	-
		CCT (≥20 μm)	44.0% (11/25)	-
	Post-PKP	BCVA (≥0.1 logMAR)	30.0% (3/10)	-
		CCT (≥20 μm)	30.0% (3/10)	-
Global Tests	All Groups	CCT (ANOVA)	Significant	<0.001
		BCVA (Kruskal-Wallis)	Significant	0.003
		ECC (ANOVA)	Not significant	0.892
Sensitivity Analysis	Post-Cataract Surgery	CCT (μm)	-29.12 (95% CI: 25.34–32.90)	<0.001
		BCVA (logMAR)	-0.25 (95% CI: 0.20–0.30)	0.001
	FECD	CCT (μm)	-24.15 (95% CI: 19.78–28.52)	<0.001
		BCVA (logMAR)	-0.15 (95% CI: 0.10–0.20)	0.003
	Post-DMEK	CCT (μm)	-7.89 (95% CI: 4.12–11.66)	0.010
		BCVA (logMAR)	-0.14 (95% CI: 0.08–0.20)	0.015
	Post-PKP	CCT (μm)	-6.50 (95% CI: -22.34 to 9.34)	0.389
		BCVA (logMAR)	-0.06 (95% CI: -0.24 to 0.12)	0.502*

CCT: central corneal thickness, BCVA: best-corrected visual acuity, logMAR: logarithm of the minimum angle of resolution, ECC: endothelial cell count, FECD: Fuchs endothelial corneal dystrophy, DMEK: Descemet membrane endothelial keratoplasty, PKP: penetrating keratoplasty, ANOVA: analysis of variance.

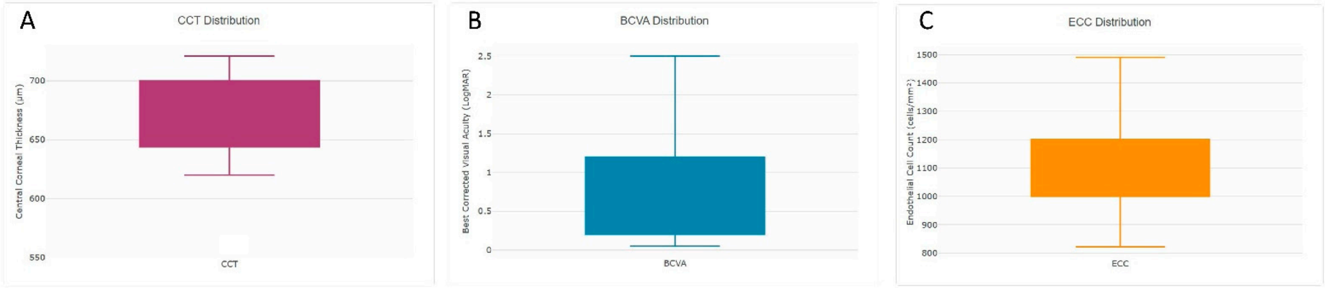

Figure S1: Visual assessments of data distribution in the PKP group (n=10). (A) Box plot for BCVA, confirming non-normality (Shapiro-Wilk p=0.03). (B) Box plot for CCT, indicating normality (p=0.12). (C) Box plot for ECC, indicating normality (p = 0.15).

p-values for BCVA in the PKP group were calculated using non-parametric tests due to non-normal distribution. Bonferroni-corrected threshold for significance is p < 0.00417 for regression and global tests.
